# Effects of acute bouts of evening resistance or endurance exercises on sleep EEG and salivary cortisol

**DOI:** 10.3389/fphys.2024.1313545

**Published:** 2024-01-23

**Authors:** Joy Perrier, Antoine Langeard, Chandrou Koumar Ouma, Bruno Sesboüé, Patrice Clochon, Jean-Noël Prevost, Françoise Bertran, Damien Davenne, Nicolas Bessot

**Affiliations:** ^1^ Normandie University, UNICAEN, PSL Université, EPHE, INSERM, U1077, CHU de Caen, GIP Cyceron, Neuropsychologie et Imagerie de la Mémoire Humaine, Caen, France; ^2^ Normandie University, UNICAEN, INSERM, GIP Cyceron, UMR-S 1075—Mobilités: Vieillissement, Pathologie, Santé—Comete, Caen, France; ^3^ Department of Clinical Physiology, CHU de Caen, Caen, France

**Keywords:** arousal, sleep architecture, endurance exercise, resistance exercise, sleep spectral analysis, exercise

## Abstract

**Introduction:** Deleterious effects of exercise close to bedtime could be due to increased physiological arousal that can be detected during sleep using sleep spectral analysis. Resistance and endurance exercises have different effects on cortisol release that may lead them to impact sleep spectral signatures differently. The present study aimed to investigate the effects of two types of evening exercise on sleep architecture, sleep spectral parameters and salivary cortisol.

**Methods:** Young healthy participants came to our laboratory to undergo 3 counterbalanced pre-sleep conditions that started 1 h before bedtime (a resistance and an endurance exercise conditions of 30 min duration, identical in terms of workload; and a control condition) followed by polysomnographic recordings. Results were compared between the three conditions for 16 participants.

**Results:** Sleep efficiency was lower after both endurance and resistance exercise than after the control condition. Total sleep time was lower after endurance exercise compared to the control condition. Sleep spectral analyses showed that both endurance and resistance exercises led to greater alpha power during N1 sleep stage and greater theta power during N2 sleep stage compared to the control condition. The endurance exercise led to greater beta power during N2 sleep stage, greater alpha power during REM sleep, and higher cortisol levels compared to the control condition (trend), and compared to the resistance exercise condition (significant). The resistance exercise led to lower beta power during N2 sleep stage than the control condition and lower cortisol levels than the endurance exercise condition.

**Discussion:** This study underlines significant modifications of sleep quality and quantity after both moderate evening endurance and resistance exercises. Still, these effects cannot be considered as deleterious. In contrast to the resistance exercise, endurance exercise led to an increase in sleep EEG activity associated with hyperarousal during sleep and higher cortisol levels, suggesting an hyperarousal effect of endurance exercise performed in the evening. These results align with previous warning about the arousal effects of evening exercise but do not support the notion of deleterious effects on sleep. While these results provide support for the physiological effects of evening exercises on sleep, replication with larger sample size is needed.

## 1 Introduction

Regular bouts of physical activity are recommended (Stepanski and Wyatt, 2003), notably to improve mental and physical health including sleep (Yang et al., 2012; Chennaoui et al., 2015; Taheri et al., 2023). However, these recommendations also warn that exercise close to bedtime should be avoided as it is speculated that it could disrupt sleep (Hauri, 1968). To our knowledge, there is a lack of information to support the latter. In particular, most previous studies have failed to highlight the deleterious effects of evening exercise on sleep (Myllymäki et al., 2011; Flausino et al., 2012; Buman et al., 2014; Kredlow et al., 2015; Mnguni et al., 2023). A recent review coupled to a meta-analysis has analyzed results from almost 30 studies respectively with the aim to compare the effects of different intensities of acute evening exercise on sleep in healthy adults. Results revealed that, regardless of intensity, acute evening exercise completed before bedtime did not disrupt subsequent sleep (Yue et al., 2022). The review and meta-analysis from Frimpong and others did not show deleterious effects of regular evening exercise of high intensity, while acute evening exercise of high intensity reduced rapid eye movement sleep (Frimpong et al., 2021). The study by Oda and Shirakawa suggested that an exercise of high intensity performed in the evening led to delayed sleep onset as well as shortened total sleep time and decreased sleep efficiency, while evening exercise of moderate intensity did not (Oda and Shirakawa, 2014). Seemly, Saidi and others investigated the effects of afternoon compare to evening exercise in young adolescent athletes and has shown that evening exercise led to lower sleep efficiency and longer sleep onset latency. Such effects were also moderated by chronotype (Saidi et al., 2023). Therefore, the effects of acute exercise performed in the evening is still controversial. One hypothesis that has been proposed is that evening exercise leads to an increased physiological excitement (hyperarousal) (Oda and Shirakawa, 2014).

Physiological arousal can be evaluated using salivary cortisol as a biomarker of the hypothalamic-pituitary-adrenal (HPA) axis’s activity (Törnhage, 2009) in response to physical stress (Hellhammer et al., 2009). In particular, acute exercise activates the HPA axis leading to an increase in cortisol release (Theron et al., 1984; Strassman et al., 1989; Chen et al., 2017) that differs according to the exercise type (Schwarz and Kindermann, 1990; Hackney et al., 1995; Anderson et al., 2016). Indeed, previous studies have mainly shown larger cortisol increases following endurance exercises compared to resistance exercises. Schwarz and Kindermann (Schwarz and Kindermann, 1990) showed that a maximum endurance exercise (incrementally graded exhaustion exercise) led to an increase in adreno-corticotropic hormone of approximately twice as high as a resistance exercise (1-min exercise). Anderson and others (Anderson et al., 2016) reported a large increase in cortisol concentration following an endurance exercise. Thus, endurance exercise seems to induce higher cortisol levels than resistance exercise. We can specifically expect arousal effects of evening exercise and we may predict that an endurance exercise can lead to higher arousal as assessed by salivary cortisol than a resistance exercise.

Spectral analyses of the sleep electroencephalogram (EEG) can also be used to further evaluated cortical activity related to either arousal, environment awareness or cortical excitation during sleep. However, for now, information is missing about the effects of exercises close to bedtime on sleep spectral parameters. Especially, cortical arousal is related to the power spectrum in the beta EEG band during Non-Rapid Eye Movement - NREM sleep (i.e., Sleep stages N2 and N3) with an increased beta power being linked with increased arousal (Törnhage, 2009; Feige et al., 2013). Increased alpha power in the alpha EEG band has been suggested to represent higher environmental awareness (McKinney et al., 2011; Simor et al., 2013) during N1 sleep stage. During N2 sleep stage, this alpha band has been related to either cortical excitation (Cantero et al., 2002) or cortical inhibition (Benca et al., 1999) during REM sleep. The role of alpha oscillations during REM sleep is thus still a matter of debate.

The current study aimed at investigating the effects of two types of evening exercise using a more finely-tuned investigation of sleep (i.e., sleep spectral analyses) and cortisol investigations. Our hypothesis was that evening exercise, especially an endurance one, may lead to increased physiological and cortical arousal compare to a control condition of no exercise. Such hyperarousal would be accompanied with lower sleep quantity and/or quality. Our results are expected to provide support for health recommendations warning of arousal effects of evening exercise.

## 2 Materials and methods

### 2.1 Participants

19 young adult athletes, 15 males and 4 females, (21–27 years old, 22.9 ± 1.5 years) participated with informed consent. However, due to bad quality of the EEG signal, 3 participants were discarded from the analyses. Results below are reported for 16 participants (12 males and 4 females; 20.9 ± 1.6 years). All participants were free of medication known to affect sleep or the circadian system, cardiovascular medication, or psychotropic medication. Participants were excluded if they: (1) had current or past dependence on alcohol, opiates, benzodiazepines, or any illicit drugs; (2) smoked more than five cigarettes per day; (3) drank more than 28 units of alcohol per week; (4) consumed more than 150 mg of caffeine per day; (5) had a mean bedtime not falling between 09:30 PM and 12:30 AM. The chronotypes of the participants were either indifferent type or moderate morning types (Horne and Ostberg, 1976). Subjective sleep quality was assessed using the Pittsburg Sleep Quality Index (Buysse et al., 1989). This study was approved by the local ethics committee. Participant characteristics are described in Table 1.

**TABLE 1:** Participants’ characteristics (Mean ± SD).

Characteristics	
Sex	4 women
	12 men
Age (years)	20.9 ± 1.6
Weight (kg)	69 ± 10.63
Body Mass Index (kg/m^2^)	22.146 ± 3.09
Physical activity (hours/week)	9.47 ± 562
Chronotypes	14 indifferent type
	4 moderate morning type
Subjective sleep quality (A.U.)	5 ± 1.73
Usual mean bedtime (hh:mm)	23:07 ± 00:12
Usual mean wake time (hh:mm)	08:09 ± 00:32

The chronotypes are given following results of the Horne and Otstberg questionnaire (Horne and Ostberg, 1976). Subjective sleep quality was assessed using the Pittsburg Sleep Quality Index (Buysse et al., 1989). Usual bedtime and wake time were assessed during 15 days using actigraphy for each participant.

### 2.2 General procedure

Each subject performed 3 polysomnographic recordings on 3 separate nights with 3 different pre-sleep conditions (resistant exercise, endurance exercise, control condition). The 3 sessions were counterbalanced and were separated by 7 days. The control condition consisted of being rest, sitting on a chair. Participants went to bed at 11.00 PM and were awakened at 7.00 AM for each condition and were instructed to not perform exercise of any intensity the day of each session. Mean activity the day before each session was assessed with actigraphy and did not differ significantly between conditions (*p* = 0.52).

### 2.3 Exercise characteristics

The exercise started at 10:00 PM and was performed for 30 min. The resistance exercise consisted of 72 bouts of 5 s of pedaling (5 revolutions of wheel: gear ratio = 52/14) followed by 20 s of rest on a cyclo-ergometer at a workload of 5.89 × body weight (kg) + 27.54 W (power to overcome braking torque + inertia). The endurance exercise consisted of 30 min of pedaling (60 rev. min-1) at a workload of 1.18 × body weight (kg) + 5.51 W. The resistance and endurance exercises were identical in total external work (2122 × body weight (kg) + 9915 J). Our protocol was built based on body mass of each participant with a fixed workload. One may argue that such protocol not tailored to the fitness level of the participants could lead to different levels of exercise difficulty. The rationale for using an approach based on body mass was chosen in order to avoid large variability of temperature increases that is known to impact sleep physiology (Oda and Shirakawa, 2014; Kredlow et al., 2015). The use of a percentage of maximal aerobic power would have induced a large disparity in power by kilos of muscle involved for each subject. Thermal response to exercise should more linked to the power/body mass ratio than percentage of maximal aerobic power.

### 2.4 Assessments

#### 2.4.1 Salivary cortisol measurement

Saliva samples were collected just after exercising according to previous recommendations related to salivary cortisol measurement following exercise (Powell et al., 2015). Participants placed a sterile cotton swab (Salivette, Sarstedt AG and Co., Germany) in their mouth, chewed it for 1 min, and placed the saturated swab into a plastic vial. Samples were centrifuged for 4 min at 2500 rpm to remove the saliva from the swab. Saliva was stored at +4°C and assayed within 5 days. Cortisol concentrations were assayed in duplicate by enzyme-linked immunosorbant assay (ELISA, Cortisol: Salimetrics. State college PA). Standards (cortisol 1.8–0.007 μg/dL) were assayed in the same run. Samples were quantified by colorimetric analysis at 450 nm.

#### 2.4.2 Sleep assessments and architecture

Sleep was recorded in the laboratory using an ambulatory polysomnography (PSG) monitor (Medatec Dream). A standard montage of PSG was used and included EEG channels (F3, F4, C3, C4, O1, O2, T3, T4, referenced on A1 and A2, two electro-oculogram (EOG) channels, and one submental electromyogram (EMG) channel. The setup was complemented by recordings from the left and right anterior tibialis muscle, recordings of nasal/oral airflow, thoracic and abdominal effort, body position, and oximetry. All PSG were scored in a double blinded manner according to the standard criteria (Berry et al., 2012) by experienced sleep specialists.

Total sleep time (TST), sleep efficiency (SE), sleep onset latency, percentages of sleep stages, and number of awakenings after sleep onset (WASO) were quantified. Sleep onset latency was defined as the time between lights-off and the first epoch of N2 sleep stage. SE was calculated as TST divided by time in bed and multiplied by 100.

#### 2.4.3 Sleep spectral analyses

The EEG data were filtered with a band pass filter of 0.16–70 Hz and sampled at 200 Hz. Computerized spectral analysis was performed with Fast Fourier Transformation (FFT) on the filtered EEG after visual elimination of epochs with artifacts (eye-movement, electrocardiogram, electromyogram, or movement-related artifacts). Spectral analysis was performed on 5.12-s epochs on the mean between C3 and C4 electrodes (as central electrodes are known to capture most of the cortical activity and because we did not have any *a priori* area-specific hypotheses) referenced on linked mastoid A1 and A2. Before computing the FFT, the data were tapered with the Hamming window. The FFT was computed on artifact-free epochs and was determined for each sleep stage using the total number of epochs corresponding to the maximum number of artifact-free epochs observed in all subjects. The relative power was calculated by dividing the absolute power in each frequency band by the total power of the whole spectrum. The relative power spectrum is reported as the power spectral densities in W/Hz in the following frequency bands: theta (4–7.5 Hz); alpha (7.5–12.5 Hz); sigma (12.5–14 Hz); and beta (14–30 Hz). All night power averages were obtained separately for NREM N1, N2, N3 sleep stages, and REM sleep stage. The use of relative power has been recommended in previous EEG studies (Nuwer, 1988; Hot et al., 2011). We preferred using relative power rather than absolute power because the former is not affected by the electrical properties of the head volume conductor. In addition, we wanted to highlight (among other) beta EEG band modifications in relationship with previous findings in insomnia patients and arousal state, that explains why we choose sleep spectral analysis instead of other sleep microstructure investigation methods.

### 2.5 Statistical analysis

Statistical analysis was done using R 3.1.2 software (www.r-project.org). To capture the session effect (endurance and resistance exercises and control sessions) on variables, we selected a linear mixed model (LME) as the methodological approach. To capture serial within-subject correlation, we investigated different correlations structures. Using the Bayesian information criteria (BIC), the time series typical AR (1) error structure was selected. Heteroscedasticity was integrated into the residual variance function. We carried out *post hoc* tests within the LME framework. To assess whether a variable had a significant effect, we followed the approach of Pinheiro and Bates (Author Anonymous, 2000) and compared models with and without the respective variable by means of a likelihood ratio test (LRT), among other secondary results (e.g., AIC, Akaike Information Criterion; BIC, Bayesian Information Criterion). Effect sizes (eta squared) were calculated using the MuMIn package in R following the approach of Westfall and others (Westfall et al., 2014).

## 3 Results

Analyses on cortisol levels showed a main effect of condition (L.r. = 6.42; *p =* 0.040, effect size = 0.76). Post hoc tests revealed a trend for higher cortisol levels in the endurance exercise condition (2.52 ± 0.79, in µg/L) compared to the control condition (2.14 ± 0.43, in µg/L) at *p* = 0.085. The cortisol levels were also higher in the endurance exercise condition compared to the resistance exercise condition (2.08 ± 0.46, in µg/L) at *p* = 0.038. The resistance exercise and the control conditions did not differ significantly (*p* = 0.94).

Sleep architecture analyses revealed a main effect of condition on SE (L.r. = 9.22; *p* = 0.027) and TST (L.r = 12.01; *p* = 0.017). Post hoc tests showed lower SE (*p* < 0.001) in both the resistance and the endurance exercise conditions compared to the control condition and a trend for lower TST in the endurance exercise condition (*p* = 0.079) compared to the control condition. The two exercise conditions did not differ significantly for SE (*p* = 0.10), and the resistance exercise condition did not differ from both the endurance (*p* = 0.65) and the control (*p* = 0.48) ones for TST. None of the other sleep parameters from the sleep architecture investigation led to significant results (see Table 2).

**TABLE 2:** Sleep architecture results (Mean ± SD).

	Control	Endurance exercise	Resistance exercise	*p-*values condition effect	Effect sizes
**Sleep onset latency, minutes**	14.43 ± 7.30	21.46 ± 13.26	21.53 ± 11.58	0.24	-
**Sleep efficiency, %**	93.34 ± 2.39	90.49 ± 4.77	91.05 ± 5.10	**0.027**	**0.56**
**Total sleep time (TST), minutes**	412 ± 19.89	394 ± 36.29	406 ± 30.72	0.017	**0.41**
**Number of WASO (A.U.)**	4.45 ± 3.04	3.40 ± 2.67	4.40 ± 3.11	0.63	-
**Duration of WASO (min)**	5.91 ± 3.70	5 ± 3.50	3.86 ± 3.61	0.31	-
**Wake duration (min)**	26.54 ± 12.52	39.25 ± 19.93	37.29 ± 29.87	0.056	-
**NREM N1 sleep stage % of TST**	3.95 ± 3.37	5.586 ± 5.239	3.24 ± 3.02	0.22	-
**NREM N2 sleep stage % of TST**	49.32 ± 9.58	51.84 ± 7.94	54.93 ± 8.37	0.19	-
**NREM N3 N2 sleep stage % of TST**	26.64 ± 5.29	26.21 ± 4.546	28.20 ± 4.53	0.46	-
**REM sleep stage % of TST**	17.59 ± 7.85	16.11 ± 6.038	13.55 ± 7.02	0.34	-

All conditions, endurance and resistance exercises and control conditions (without any exercise). To make the table easier to read, only *p*-values related to the model coefficients and *post hoc* results are reported here. For more statistical information related to the model comparisons (LRT), see the Results section. The bold values represent *p*<0.05 for the condition effect. SD: standard deviation; min: minutes, REM: rapid eye movement, WASO: wake after sleep-onset.

Results of sleep spectral analysis are reported in Table 3. During N1 sleep stage, a main effect of condition was found for the alpha band (L.r. = 16.27; *p* = 0.037). Post hoc results showed that both endurance and resistance exercise conditions led to higher alpha power than the control condition during N1 sleep stage (*p* = 0.007 and *p* = 0.033, respectively).

**TABLE 3:** Power spectral density results (Mean ± SD, W/Hz).

Sleep stages	Frequency bands	Control	Endurance exercise	Resistance exercise	*p-*values condition effect	Post hoc results between conditions			Effect sizes
						End vs. Resist	End vs. Con	Resist vs. Con	
**NREM N1 sleep stage**	Alpha	17.17 ± 4.67	21.96 ± 7.71	21.18 ± 8.03	**0.027**	0.99	**0.0067**	**0.033**	**0.70**
	Beta	17.37 ± 6.34	17.07 ± 6.11	18.46 ± 7.08	0.13	-	-	-	-
	Theta	21.40 ± 4.52	21.45 ± 4.45	20.26 ± 2.99	0.35	-	-	-	-
	Sigma	3.14 ± 1.07	2.97 ± 0.60	3.51 ± 1.58	0.065	-	-	-	-
**NREM N2 sleep stage**	Alpha	14.57 ± 3.22	15.16 ± 3.17	15.12 ± 2.76	0.18	-	-	-	-
	Beta	13.98 ± 4.06	14.09 ± 5.16	13.52 ± 4.43	**0.037**	0.84	**0.025**	**0.046**	**0.48**
	Theta	21.27 ± 1.91	22.39 ± 2.97	22.95 ± 2.56	**0.0058**	0.87	**0.022**	**0.0031**	**0.28**
	Sigma	5.40 ± 2.87	5.36 ± 2.43	5.80 ± 3.05	0.94	0.38	0.99	0.37	-
**NREM N3 sleep stage**	Alpha	8.41 ± 2.73	10.49 ± 3.44	9.18 ± 3.00	0.25	-	-	-	-
	Beta	6.19 ± 2.42	5.53 ± 2.51	5.97 ± 3.27	0.45	-	-	-	-
	Theta	17.49 ± 2.30	17.67 ± 1.96	18.09 ± 1.81	0.64	-	-	-	-
	Sigma	2.32 ± 1.25	2.08 ± 0.61	2.72 ± 1.45	**0.0025**	0.11	0.67	0.54	0.34
**REM sleep stage**	Alpha	15.08 ± 4.84	17.67 ± 4.92	16.47 ± 4.84	**0.011**	0.13	**0.0026**	0.201	**0.39**
	Beta	14.85 ± 5.07	14.19 ± 4.78	13.32 ± 4.91	0.31	-	-	-	-
	Theta	25.37 ± 4.25	26.30 ± 3.42	26.30 ± 3.46	0.54	-	-	-	-
	Sigma	2.75 ± 0.80	2.78 ± 0.72	2.86 ± 0.88	0.39	-	-	-	-

All conditions, endurance and resistance exercises and control conditions (without any exercise). To make the table easier to read, only *p*-values related to the model coefficients and *post hoc* results are reported here. For more statistical information related to the model comparisons (LRT), see the Results section. The bold values represent *p*<0.05 for the condition effect. SD: standard deviation, REM: rapid eye movement; End: Endurance exercise; Resist: Resistance exercise; Con: Control condition.

DuringN2 sleep stage, a main effect of conditions was found for the beta band (L.r. = 8.47; *p* = 0.0027) and the theta band (L.r. = 10.31; *p* = 0.0058). Post hoc results revealed higher beta power in the endurance exercise condition (*p* = 0.025) and lower beta power in the resistance exercise condition (*p* = 0.046) compared to the control condition. Results showed higher theta power in both endurance (*p* = 0.022) and resistance (*p* = 0.0031) exercises conditions compared to the control condition. No main effect of conditions was found for the sigma band (L.r. = 0.85; *p* = 0.94).

DuringN3 sleep stage, no significant effect of conditions was found following *post hoc* tests for any of the bands considered.

During REM sleep, a main effect of conditions was found for the alpha band (L.r. = 11.15; *p* = 0.011). Post hoc results showed increased alpha power in the endurance exercise condition compared to the control condition (*p* = 0.0027); resistance exercise condition results did not differ from the control condition (*p* = 0.20).

## 4 Discussion

The current investigation aimed at investigating the effects of two types of evening exercise using a more finely-tuned investigation of sleep (i.e., sleep spectral analyses) and cortisol investigations. Our hypothesis was that evening exercise, especially an endurance one, may lead to increased physiological and cortical arousal compare to a control condition of no exercise. Such hyperarousal would be associated with lower sleep quantity and/or quality.

The results reported here show that moderate intensity exercises (both endurance and resistance) completed 30 min before bedtime were associated with lower sleep quality and quantity than a control condition with no exercise, although such effects were minor and cannot be considered as deleterious. Results also showed that, in contrast to the resistance exercise, endurance exercise led to an increase in EEG activity associated with hyperarousal during sleep and higher cortisol levels, suggesting an hyperarousal effect of endurance exercise performed in the evening. These results align with those of Oda and Shirakawa (Oda and Shirakawa, 2014) suggesting that pre-sleep exercise cause a physiological excitement. Results are discussed in details below.

Both endurance and resistance exercises led to sleep architecture small modifications. SE was modified following both endurance and resistance exercises with slight effects (−2% and −3% of SE following, respectively aa resistance and an endurance exercise). TST also tended to be lower following endurance exercise. Our results align with recent findings from Saidi and others (Saidi et al., 2023) showing lower sleep efficiency (−1.50%) following high intensity evening exercise, although the intensity was moderate in the current study. Both endurance and resistance exercise conditions also led to greater theta power compared to the control condition during N2 sleep stage. As the theta frequency band is known to be related to memory processes (Boyce et al., 2016), our theta power results may reflect cognitive process modulation following exercise, supporting the growing evidence of cognitive ability improvement following exercise (see (Tomporowski, 2003; Gomez-Pinilla and Hillman, 2013; Hötting and Röder, 2013), for reviews). However, this hypothesis is speculative because no memory task was performed in the current protocol. Both the resistance and the endurance exercises also led to greater alpha power during N1 sleep stage following. During N1 sleep stage, the alpha band has been suggested to represent environmental awareness (McKinney et al., 2011; Simor et al., 2013). These results thus suggest greater environmental awareness following evening exercises of moderate intensity, both endurance and resistance.

In contrast to the resistance exercise condition, the endurance exercise one led to both a greater beta power spectrum during N2 sleep stage, and higher salivary cortisol concentrations compared to the control condition. Both higher salivary cortisol and higher power spectrum in the beta EEG band have been related with hyperarousal (Törnhage, 2009; Feige et al., 2013). Thus, these results align with the hypothesis about greater arousal following endurance exercise (Hauri, 1968) and support previous health recommendations warning about arousal effects of evening exercise. Importantly, such arousal did not affect sleep architecture and cannot be considered as deleterious for sleep. The endurance exercise condition also led to increased alpha power during REM sleep compared to the control condition. Two main hypotheses have been proposed regarding the role of alpha oscillations during REM sleep, one related to cortical excitation (Cantero et al., 2002) while the other is related to cortical inhibition (Benca et al., 1999). Cantero and others (Cantero et al., 2002) suggested that an increased alpha power during REM sleep constitutes short periods of sleep instability (micro-arousals). However, Benca and others (Benca et al., 1999) proposed that the alpha band during REM sleep represents cortical deactivation, resulting in protection of sleep by preventing awakenings (Benca et al., 1999; Simor et al., 2013). Our results are more in favor of the latter hypothesis given that sleep architecture was not affected following the endurance exercise condition.

This study suffers from several limitations. One limitation of our study is the small sample size particularly affecting some statistical results that are nearly significant. Moreover, the effect of sex (Mong and Cusmano, 2016) and other physiological parameters could not be taken into account, as for melatonin and temperature. Regarding the latter, our protocol was built based on body mass of each participant with a fixed workload in order to avoid large variability of temperature increases that is known to impact sleep physiology (Myllymäki et al., 2011; Feige et al., 2013). Future studies should considerer assessing both temperature and melatonin in addition to cortisol. In addition, participants were young physically active adults and results could be different in elderly people or persons with a sedentary lifestyle, given that acute and regular evening exercises do not affect sleep similarly (Frimpong et al., 2021). Finally, we were not able to provide the exact time of completion for habitual physical activity of participants and results may be different if participants are used to perform exercise in the evening or not. We acknowledge that future studies in the field are needed.

## 5 Conclusion

Using sleep spectral analysis and salivary cortisol measures, this study provides support for both cortical and physiological arousal effects of an endurance exercise of moderate intensity performed in the evening, while a resistance exercise did not. These results provide additional knowledge about the physiological effects of evening exercise and align with previous warning of arousal effects of evening exercise. However, the current results do not support the notion according to such hyperarousal is associated with deleterious effects on sleep architecture. Therefore, it is here suggested that an evening exercise of moderate intensity does not disrupt sleep, although it is associated with physiological and cortical excitement. Further investigations are needed in other populations such as elderly people or persons with a sedentary lifestyle as well as with larger sample sizes.

## Data Availability

The raw data supporting the conclusion of this article will be made available by the authors, without undue reservation.
